# Reproducible protocols for metagenomic analysis of human faecal phageomes

**DOI:** 10.1186/s40168-018-0446-z

**Published:** 2018-04-10

**Authors:** Andrey N. Shkoporov, Feargal J. Ryan, Lorraine A. Draper, Amanda Forde, Stephen R. Stockdale, Karen M. Daly, Siobhan A. McDonnell, James A. Nolan, Thomas D.S. Sutton, Marion Dalmasso, Angela McCann, R. Paul Ross, Colin Hill

**Affiliations:** 1 APC Microbiome Institute, University College Cork, Cork, Ireland; 2 Department of Food Biosciences, Teagasc Food Research Centre, Moorepark, Fermoy, Co. Cork Ireland; 3 Normandie Univ, UNICAEN, EA4651 ABTE, F-14032 Caen, France

**Keywords:** Human gut microbiome, Phageome, Virome, Bacteriophage, Metagenomics

## Abstract

**Background:**

Recent studies have demonstrated that the human gut is populated by complex, highly individual and stable communities of viruses, the majority of which are bacteriophages. While disease-specific alterations in the gut phageome have been observed in IBD, AIDS and acute malnutrition, the human gut phageome remains poorly characterised. One important obstacle in metagenomic studies of the human gut phageome is a high level of discrepancy between results obtained by different research groups. This is often due to the use of different protocols for enriching virus-like particles, nucleic acid purification and sequencing.

The goal of the present study is to develop a relatively simple, reproducible and cost-efficient protocol for the extraction of viral nucleic acids from human faecal samples, suitable for high-throughput studies. We also analyse the effect of certain potential confounding factors, such as storage conditions, repeated freeze-thaw cycles, and operator bias on the resultant phageome profile. Additionally, spiking of faecal samples with an exogenous phage standard was employed to quantitatively analyse phageomes following metagenomic sequencing. Comparative analysis of phageome profiles to bacteriome profiles was also performed following 16S rRNA amplicon sequencing.

**Results:**

Faecal phageome profiles exhibit an overall greater individual specificity when compared to bacteriome profiles. The phageome and bacteriome both exhibited moderate change when stored at + 4 °C or room temperature. Phageome profiles were less impacted by multiple freeze-thaw cycles than bacteriome profiles, but there was a greater chance for operator effect in phageome processing. The successful spiking of faecal samples with exogenous bacteriophage demonstrated large variations in the total viral load between individual samples.

**Conclusions:**

The faecal phageome sequencing protocol developed in this study provides a valuable additional view of the human gut microbiota that is complementary to 16S amplicon sequencing and/or metagenomic sequencing of total faecal DNA. The protocol was optimised for several confounding factors that are encountered while processing faecal samples, to reduce discrepancies observed within and between research groups studying the human gut phageome. Rapid storage, limited freeze-thaw cycling and spiking of faecal samples with an exogenous phage standard are recommended for optimum results.

**Electronic supplementary material:**

The online version of this article (10.1186/s40168-018-0446-z) contains supplementary material, which is available to authorized users.

## Background

The last two decades have seen tremendous progress in understanding the structure, dynamics and physiological significance of diverse microbial communities populating the human gastro-intestinal tract (GIT), collectively known as the gut microbiome. The majority of the microbial biomass of faeces is represented by bacteria (~ 10^13^ bacterial cells with a total wet mass of 0.2 kg [1]), and so, it is not surprising that the bacterial component of the microbiome has received most attention over the years and has overshadowed the study of archaea, fungi, protists and viruses infecting both eukaryotic and prokaryotic cells [2–4]. Numerous studies have shown that gut microbial communities are highly complex and highly divergent between individuals, but with a large degree of resilience and stability over time [5, 6]. Studies in germ-free mice have confirmed that gut microbial populations are important for normal development and functioning of the mammalian GIT. Various perturbations of the healthy microbiome have been associated with a large list of human diseases including inflammatory bowel disease (IBD), metabolic disorders and cancer [7].

High-throughput sequencing (HTS) technologies have revolutionised the investigation of the microbiome in recent years and have facilitated the detection, isolation and characterisation of a vast array of previously uncultured microorganisms [8–11]. Evidence suggested that viruses, albeit representing only a tiny fraction of microbiota by their combined biomass, might in fact be present at numbers comparable to those of cellular microbial symbionts [12, 13]. In earlier metagenomic HTS studies of the microbiome, ~ 6% of normalised sequencing reads and ~ 5% of the clusters of orthologous groups of genes (COGs) were reported to be of viral, mostly bacteriophage-related, origin [14, 15]. The actual numbers of viral particles per gram of faeces, however, remain a matter of controversy, with estimates ranging from 10^8^ to 10^10^ depending on the extraction method used [16, 17]. Mucosal samples were reported to contain higher viral loads [18] with the phage-to-bacteria ratio reaching 20:1 [19, 20].

Focused metagenomic HTS studies of human faecal samples enriched for virus-like particles (VLPs) identified 35–2800 phage virotypes per individual [21, 22]. Of these, the vast majority cannot be reliably linked to any particular host bacterium [23] and ~ 93% lack genetic features allowing them to be assigned to a known family according to the classification proposed by the International Committee on Taxonomy of Viruses (ICTV) [24]. Analysis of diversity between individual human gut phageome samples shows very high levels of individuality and temporal stability of gut viromes/phageomes which is concordant with the individuality and temporal stability of gut bacteriomes [21, 23]. A recent study has demonstrated, however, that within-sample diversity of phage types in the gut is quite limited with as little as 100 phage genomes on average representing 75% of normalised reads [24].

A number of recent studies have highlighted disease-specific alterations of the gut phageome in such various human diseases as IBD [25–27], acute malnutrition [28] and AIDS [29]. In the light of these studies and a rapidly growing interest in the human gut phageome in health and disease, it becomes vitally important to perform a critical assessment of currently used protocols and approaches of metagenomic HTS-based analysis of gut phageomes and to identify possible confounding factors [30]. In addition, there are several major hurdles in metagenomic analysis of the human gut phageome that need to be resolved. In particular, conflicting data has been reported by different groups on the qualitative and quantitative composition of “healthy” human gut phageomes depending on the protocol used. Published phageome datasets are often compromised by variable levels of bacterial sequence contamination [31]. Also, many of the reported methods for VLP extraction include protocols (such as CsCl density gradient centrifugation and tangential flow filtration) that are laborious, poorly reproducible, require expensive equipment and are not compatible with a high-throughput.

The majority of recently published viral metagenomic studies of the human gut and other body sites have used similar approaches for enrichment of viral particles from biological samples and to remove large amounts of contaminating DNA and RNA of bacterial and eukaryotic origin (Additional file 1: Table S1). Typically, samples are homogenised in a suitable buffer with or without the use of bead-beating, then subjected to rounds of low-speed centrifugation and filtration through a 0.2–0.45-μm pore membrane for removal of human and microbial cells, and other particulate matter [32]. Larger filter pore sizes (0.8-μm pore size) tend to introduce less bias due to retention of large and enveloped viruses, but result in greater contamination with bacterial cells. Viral particles are then concentrated from crude filtrates by means of polyethylene glycol (PEG) precipitation, dead-end filtration or tangential flow (TFF) filtration [17, 32]. Chloroform extraction of suspension of viral particles at this stage can help to remove remaining bacterial cells at the expense of losing some of the chloroform-sensitive viruses. Subsequently, the viral particles are treated with DNase and RNase to remove free nucleic acids and are then further purified by density gradient centrifugation in solutions with step- or continuous gradient concentrations of CsCl [33, 34]. The latter step results in extremely pure viral preparations; however, it is very laborious, poorly reproducible and has the potential to introduce significant bias due to loss of viruses with atypical densities.

Many published human viral metagenomic studies predominantly focus on DNA-containing viruses and do not include a reverse transcription step. In addition, they routinely include a form of whole-genome amplification (WGA) technology to increase total DNA yield (Additional file 1: Table S1). It is well known that WGA, in particular multiple displacement amplification (MDA) using the highly processive replicative polymerase φ29, can introduce significant bias due to preferential propagation of short circular single-stranded DNA molecules. Recently, an attempt was made to address this issue by using an alternative library construction protocol that requires minimal amounts of DNA, in either single- and double-stranded form [35].

Some of the recent studies have focused on optimising isolation of VLPs from human faecal samples and maximising yield of viral nucleic acids while reducing possible contamination with cellular DNA and RNA [32, 33]. However, a systematic analysis of other confounding factors such as sample collection mode, storage conditions, repeated freeze-thaw cycles, and operator-to-operator bias has not been reported.

With this in mind, we investigated the effects of some of the critical confounding factors believed to affect gut phageome diversity profiles. We investigated whether variations in collection, storage and handling conditions significantly influenced faecal phageome profiles, given that recent reports have shown them to affect bacteriome profiles [36–40]. We also conducted a study of operator-to-operator reproducibility, in which we asked different operators to consistently repeat the same phageome nucleic acid extraction protocol on the same faecal samples, under the same conditions but on separate occasions. In order to calibrate the ratios of taxonomic units as a routine standard in all microbiome analyses, we spiked faecal samples with exogenous phages to accurately evaluate absolute and relative abundances of endogenous phages. This is in line with recommendations made by Stämmler et al. [41].

## Results

### Design of the experiments

To investigate the relative impact of faecal sample storage conditions, repeated freeze-thaw cycles and operator-dependent bias on the outcome of gut phageome profiles, three separate and independent experiments were conducted on subsamples of faecal samples collected from 10 healthy adult volunteers and 3 adult IBD patients.

In the storage experiment, faecal samples from 4 healthy individuals were subjected to two different modes of treatment: storage at either room temperature or + 4 °C, with aliquots for faecal phageome profiling by shotgun sequencing and faecal bacteriome profiling by 16S amplicon sequencing sampled at 0, 6 and 24 h. In the freeze-thaw experiment, faecal samples from 3 healthy donors were repeatedly frozen (at − 80 °C) and thawed with aliquot removal after 1st, 3rd and 5th cycles. In addition, an aliquot from each sample was kept at − 80 °C for 17 days to mimic long-term storage conditions. In the operator reproducibility experiment, 3 trained scientists were asked to extract total and phageome DNA from 3 healthy faecal samples and 3 IBD faecal samples in duplicate with subsequent library preparation performed by a single individual.

In addition, a separate spiking experiment was conducted with samples collected from three healthy individuals wherein the lactococcal phage Q33 was introduced into the faecal homogenate at known concentrations, which allowed for quantification of the total bacteriophage loads in faecal samples.

The protocol used for extraction of viral nucleic acids from human faecal samples was based on a protocol for DNA extraction from murine faecal VLP fraction published by Reyes et al. [42]. Nonetheless, several important modifications were introduced to render the protocol more suited for human faecal samples, to enable recovery of viral genomic RNA and to increase overall nucleic acid yield and purity (OD 260/280 ratio).

**Fig. 1:**
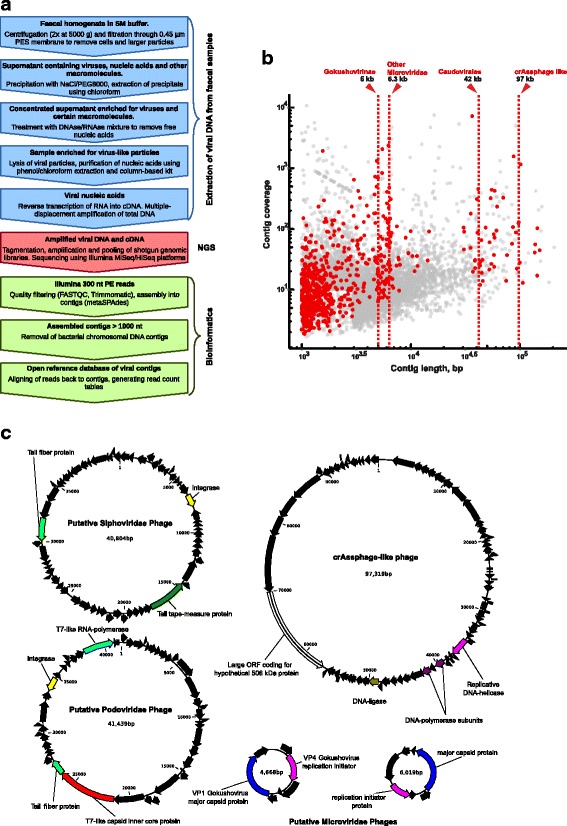
Work-flow of gut phageome metagenomic sequencing protocol used in this study and example of sequencing output. **a** Experimental and data analysis work-flow. The three main stages: sample processing, sequencing and sequencing data analyses are shown in blue, red and green, respectively. **b** Contig size and coverage statistics for the pooled contig database (*n* = 8,920) obtained in this study after size selection, *in silico* removal of potential bacterial DNA contamination, and removal of redundancy between samples. Linear contigs (*n* = 8,225) are shown in grey, circular ones (*n* = 695) in red. Centerlines for different distinct size classes of contigs are shown as dashed red lines. **c** Representative examples of different size classes of complete circular contigs identified in panel **b**. Hallmark gene characteristic of particular phage families are highlighted in colour

Briefly, the optimised protocol consisted of the following steps: (1) gentle homogenisation of faecal sample in SM buffer without the use of bead-beating; (2) separation of particulate matter by two rounds of low-speed centrifugation and filtration through 0.45-μm pore polyethersulfone (PES) membrane filters; (3) concentration of viral particles from the filtrate by PEG/NaCl precipitation; (4) removal of remaining bacterial cells and excess of PEG by chloroform extraction; (5) removal of free, capsid-unprotected nucleic acids by combined DNase/RNase treatment; (6) dissociation of viral particles using Proteinase K digestion followed by complete lysis with chaotropic salt; (7) purification of viral nucleic acids by phenol:chloroform extraction and spin-columns (Fig. 1a). Nucleic acid yields varied in a wide range from 60.5 ng to 4.3 μg (median yield 533 ng) of DNA from the same starting amount of 500 mg of faeces. Obtained samples were then subjected to reverse transcriptase treatment and MDA using φ29 polymerase with the idea to convert any co-purified RNA and ssDNA molecules into amplified dsDNA, suitable for use as a starting material in Illumina shotgun library preparation protocols.

As a result of various treatment regimens, a total of 81 shotgun libraries were sequenced using Illumina MiSeq platform (2 × 300 nt reads) producing 263,641,845 quality-filtered trimmed reads (minimal Phred score of 30, 119.27 Gb of quality-filtered sequence data) with a median read count of 3,170,798 per sample. The dataset was filtered to remove sporadic reads aligning to human genomic DNA. The extent of bacterial and eukaryotic chromosomal DNA contamination in quality-filtered and trimmed reads was estimated by aligning quality-filtered reads to a database of 16S, 23S, 18S, 28S, 5S and 5.8S rRNA sequences of bacterial, archaeal and eukaryotic origin. The median percentage of reads corresponding to ribosomal RNA genes per sample was 0.04% (interquartile range 0.06%), of which an overwhelming majority were bacterial rRNA. A few samples however, displayed elevated levels of bacterial DNA contamination reaching 0.47% of reads aligning to bacterial rRNA (Additional file 2: Table S2). We also employed alignment of reads to a database of 549–567 bp segments of a highly conserved bacterial gene *cpn60*, generally present in a single copy per genome and often employed as an alternative to 16S rRNA taxonomic marker in bacteria [43]. Median fraction of reads aligned to this gene segment was 0.00077% (IQR = 0.00182%; Additional file 3: Figure S1). To further reduce the extent of bacterial DNA contamination, contigs assembled from processed reads were filtered according to criteria described in the ‘Methods’ section.

### Assembly of phage genomic contigs

In this study, we employed an assembly-based approach instead of the more often used closed-reference database approach for classifying Illumina reads. Processed reads were assembled into contigs, individually for each sample and across all samples in the study, yielding 88,310 contigs of length greater than 1 kb. Duplicate and highly related (> 90% of identity) contigs were removed across all samples in such a way that the longer contig from each pair of homologues survived. During further processing steps, in order to select viral contigs and remove as much bacterial DNA contamination as possible, contigs were picked which fulfil at least one of the following criteria: (a) predicted as viral by Virsorter package [44]; (b) having sufficient similarity (50% identity over 90% of contig length) to known sequences in the viral section of NCBI RefSeq database [45, 46]; (c) being circular; and (d) being > 3 kb with coverage of at least 10×, and generating no alignments > 100 nucleotides (with *e* value < 1e−10) to the NCBI ‘nt’ database.

This approach resulted in an open reference database of 8920 putative viral genomic contigs ranging in size from 1000 to 207,752 bp. Six hundred ninety-five contigs (1001–151,574 bp long) were circular, suggesting that they may represent complete viral genomes, or other circular extrachromosomal DNA sequences (plasmids, conjugative transposons) inadvertently amplified by the action of polymerase φ29 (Fig. 1b). Additionally, 1057 contigs were recognised as having a viral origin by Virsorter software. One hundred sixty-one contigs (including 75 positively identified by Virsorter) could be aligned to known viral genomes in the viral section of NCBI RefSeq database. Of the latter, 157 were identified as bacteriophages, two were human papillomavirus 16 and 53 (HPV16 and HPV53), one contig was tomato yellow leaf curl virus (TYLCV) [47] and the final one was a sewage-associated gemycircularvirus-4 with unknown host range [48].

Plotting contig coverage versus length revealed the presence of distinct size classes of circular contigs (Fig. 1b). For instance, groups of similarly sized contigs likely representing small ssDNA bacteriophages of the subfamily *Gokushovirinae* (~ 5 kb) and other subfamilies within the family *Microviridae* (~ 6.3 kb) were readily identifiable [49]. In addition, a group of loosely inter-related ~ 97 kb contigs were identified with distant homologies and overall genome structure similarities to uncultured but widespread bacteriophage crAssphage (Fig. 1c).

Collectively, 49.66% of quality-filtered and trimmed Illumina reads obtained from the faecal VLP fraction across all samples in this study could be aligned to this study’s curated database of viral genomic contigs, and we omitted the remaining ~ 50% of reads from the current analysis.

### Effect of storage on faecal phageome and bacteriome composition

**Fig. 2:**
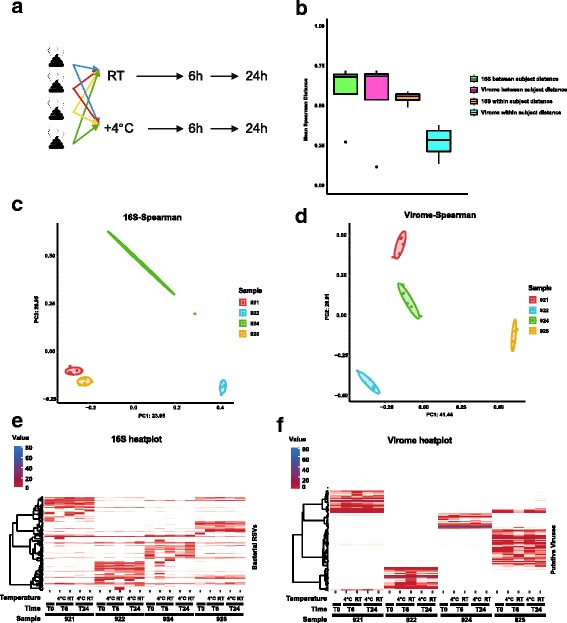
Impact of storage of faecal samples at room temperature or + 4 °C on the composition of faecal phageomes and bacteriomes. **a** Outline of experimental protocol: fresh samples were collected from 4 individuals and subjected to two modes of storage with removal of aliquots for analysis at 0, 6 and 24 h. **b** Within- and between-subject Spearman distances of relative abundance values per sample in datasets obtained using metagenomic sequencing of phageomes and 16S rRNA amplicon sequencing. **c**, **d** PCoA plots based on Spearman distances calculated for 16S rRNA amplicon and phageome datasets, respectively. **e**, **f** Heatplots of relative abundances of selected bacterial RSVs and viral contigs (> 0.5% in any of the samples) across samples collected in the experiment

Sample storage at + 4 °C or room temperature had a moderate effect on the composition of bacterial and phage components of the faecal microbiome (Fig. 2). No statistically significant variation in α-diversity, using either the Shannon index or Chao1 index, was observed between time points or treatment modes (Additional file 4: Figure S2a, b).

β-diversity analyses were conducted using Spearman rank correlation as a measure of ecological distance between samples. Both phageome and 16S ribosomal sequence variant (RSV) data strongly clustered by donor, rather than by storage mode or storage time (Fig. 2c, d; Additional file 4: Figure S2c, d). In fact, permutational ANOVA tests confirmed that most of the variation in phageomes could be explained by sample donor (*R*^2^ = 0.795, *p* = 0.001 after 1000 permutations, see Additional file 5: Table S3 for test details), while storage time had very modest, albeit statistically significant impact (*R*^2^ = 0.109, *p* = 0.001). A similar result was obtained for 16S RSV bacteriome data: *R*^2^ for donor-dependent variation was 0.553 (*p* = 0.001), and just 0.142 for time-dependent variation (*p* = 0.002).

Comparisons of mean between-subject Spearman distances, reflecting the level of individuality of faecal microbiomes, did not reveal any significant difference between phageome and 16S bacteriome data. However, the extent of variation in the qualitative and quantitative composition of phageomes reflected by average within-subject Spearman distances was significantly lower (*p* = 0.028 in Mann-Whitney *U* test) than that calculated for 16S RSV data (Fig. 2b). The latter observation suggests that the faecal phageome is more stable over time compared to the bacteriome (at least when stratified by 16S) under the storage conditions tested.

Despite certain fluctuations in the relative proportions of taxa in stored samples, DESeq2 analysis did not identify any phage sequences considered to be differentially abundant between time points and/or treatment modes (Fig. 2e, f; Additional file 4: Figure S2e, f).

### Effect of multiple freeze-thaw cycles on faecal phageome and bacteriome composition

**Fig. 3:**
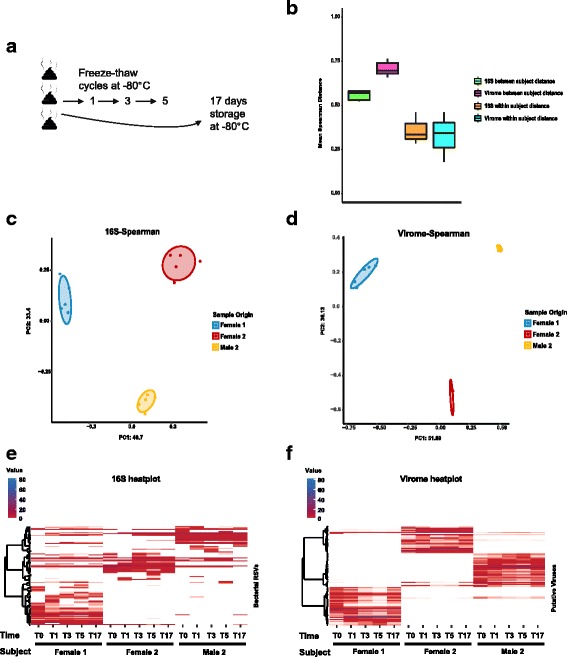
Impact of multiple freeze-thaw cycles on the composition of faecal phageomes and bacteriomes. **a** Outline of experimental protocol: fresh samples were collected from 3 individuals and subjected to 1–5 cycles of freezing at − 80 °C and complete thawing. One aliquot was kept at − 80 °C for 17 days to mimic long-term freezer storage. **b** Within- and between-subject Spearman distances of relative abundance values per sample in datasets obtained using metagenomic sequencing of phageomes and 16S rRNA amplicon sequencing. **c**, **d** PCoA plots based on Spearman distances calculated for 16S rRNA amplicon and phageome datasets, respectively. **e**, **f** Heatplots of relative abundances of selected bacterial RSVs and viral contigs (> 0.5% in any of the samples) across samples collected in the experiment

Multiple freeze-thaw cycles appeared to have only a mild effect on the composition of the phage and bacterial components of the faecal microbiome–results which were similar to that obtained for the storage experiment (Fig. 3). As indicated by Shannon index, Chao1 index and numbers of observed taxa, no statistically significant change in α-diversity could be seen between the original samples and the same samples which underwent various numbers of freeze-thaw cycles or prolonged storage at − 80 °C (Additional file 6: Figure S3a, b).

β-diversity analysis consistently clustered samples by donor subject (Fig. 3c, d; Additional file 6: Figure S3c, d): the fraction of variance explained (*R*^2^) by donor was 0.873 for the phage component (*p* = 0.001 in PERMANOVA, see Additional file 5: Table S3 for test details) and 0.81 for the 16S RSV bacterial component (*p* = 0.001). The fraction of variance explained by the number of freeze-thaw cycles was statistically insignificant in both phageome (*R*^2^ = 0.0629, *p* = 0.092) and 16S RSV data (*R*^2^ = 0.0836, *p* = 0.152).

Unlike the storage experiment, no difference in within-subject average Spearman distances could be seen between phageome and 16S RSV data. However, average inter-individual distances were considerably higher in phageome samples compared to 16S RSV bacteriome samples, which potentially reflects a higher level of faecal phageome individuality between subjects.

As in the storage experiment, no single contig could be identified as being differentially abundant in cycle 0 versus cycle 5 comparisons, despite some variations in the overall composition of the phageomes between free-thaw cycles (Fig. 3e, f; Additional file 6: Figure S3e, f).

### Operator-dependent bias in the analysis of faecal phageome and bacteriome composition

**Fig. 4:**
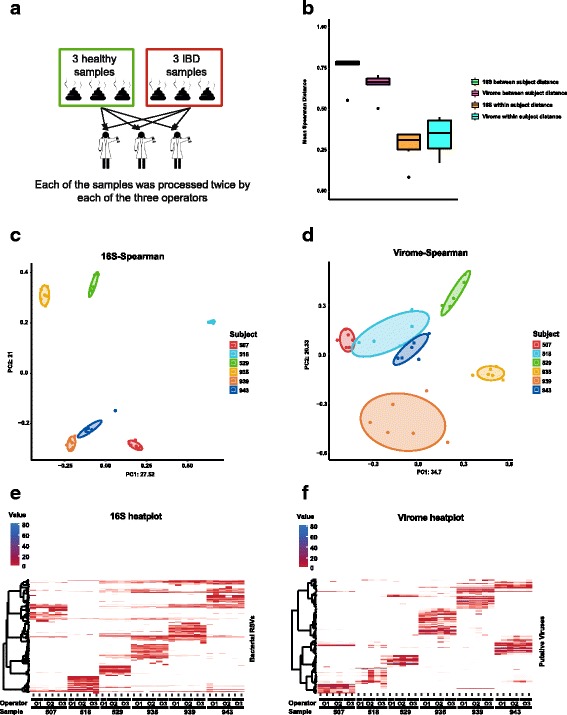
Inter-operator bias in faecal phageome and bacteriome profiling. **a** Outline of experimental protocol: fresh samples were collected from 6 individuals (3 healthy and 3 IBD patients) and processed twice each for phageome and total faecal DNA by each of the three laboratory technicians. **b** Within- and between-subject Spearman distances of relative abundance values per sample in datasets obtained using metagenomic sequencing of phageomes and 16S rRNA amplicon sequencing. **c**, **d** PCoA plots based on Spearman distances calculated for 16S rRNA amplicon and phageome datasets, respectively. **e**, **f** Heatplots of relative abundances of selected bacterial RSVs and viral contigs (> 0.5% in any of the samples) across samples collected in the experiment

The three operators introduced a certain degree of variation into the phageome data but not into the 16S bacteriome data (Fig. 4). Despite a very clear subject-specific clustering pattern of the phageome samples (*R*^2^ = 0.835, *p* = 0.001 in PERMANOVA, see Additional file 5: Table S3 for test details), a statistically significant amount of variation between samples could be explained by the operator (*R*^2^ = 0.028, *p* = 0.004, Fig. 4d; Additional file 7: Figure S4d). By contrast, almost all of the variation in 16S bacteriome profiling results could be explained by the subject of origin (*R*^2^ = 0.905, *p* = 0.001), while the amount of variation explained by operator was statistically insignificant (*R*^2^ = 0.0097, *p* = 0.129, Fig. 4c; Additional file 7: Figure S4d). In addition to that, average inter-individual Spearman distances were greater for 16S bacteriome data compared to phageome data (*p* = 0.041 in Mann-Whitney *U* test) reflecting better clustering of 16S data by subject. When mean intra-subject distances were compared between 16S and phageome data, no significant difference could be seen (Fig. 4b).

No statistically significant difference in α-diversity of 16S RSVs and phage genomic contigs was evident across the operators using either Chao1 or Shannon diversity indices. However, it was noted that there was a tendency towards a decrease in phageome Shannon diversity with two out of three operators (*p* = 0.078 in Kruskal-Wallis *H* test; Additional file 7: Figure S4a, b).

DESeq2 analysis was able to identify a total of 37 putative phage genomic contigs ranging in size from 1062 to 144,056 nucleotides, which were differentially represented in some faecal samples processed by the three operators (log2 fold change ranging from − 8.3 to + 6.8 relative to base mean according to DESeq2 test, Additional file 7: Figure S4e, f; Fig. 4e, f; Additional file 8: Table S4). This probably reflecting the noticeable amounts of operator-dependent β-diversity in the phageome dataset.

### Assessing total faecal phage loads by spiking faecal samples with an exogenous phage

**Fig. 5:**
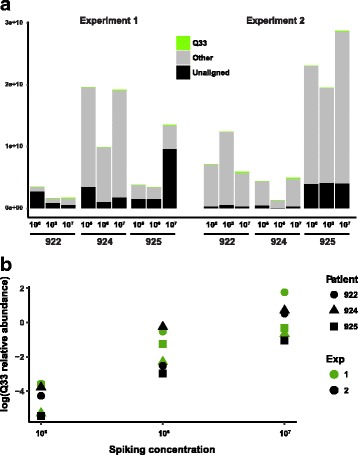
Spiking of faecal samples with an exogenous phage enables absolute quantification of faecal viruses by metagenomic sequencing. **a** Percentage of reads aligning to the spiked lactococcal bacteriophage Q33 genome depends linearly on the spiked phage titre in faecal sample. **b** Spiking of faecal samples at concentration of 10^6^ pfu g^− 1^ seems to be optimal in terms of percentage of reads aligning to the internal standard phage genome

Spiking of faecal homogenates with various amounts (10^5^, 10^6^ and 10^7^ pfu ml^− 1^) of lactococcal phage Q33 [50] resulted in a quantitative recovery of reads aligning to its genome (Fig. 5a, experiment 1). The percentage of reads aligned to the Q33 genome was a linear function (*R*^2^ of linear regression 0.73–0.99) of the amount of spiked phage. The Q33 alignment rates ranged from 0.009–0.048% when spiking was performed at a concentration of 10^5^ pfu ml^− 1^, to 0.95–14.9% when 10^7^ pfu ml^− 1^ of phage was added. Reads aligning to phage Q33 genome could be assembled into a single contig which contained 99.589% of the original Q33 genome with no miss-assemblies, 27 mismatches and 10 indels as judged by QUAST assembly quality test. In a second experiment, we spiked same three faecal samples with mixture of phage Q33 and an RNA coliphage Qβ again at concentrations of 10^5^, 10^6^ and 10^7^ pfu ml^− 1^ of each of the two phages (Fig. 5a, experiment 2). For Q33, a result very similar to experiment 1 was obtained; however, no reads aligning to Qβ phage genome could be detected in any of the spiked samples.

Total phage loads calculated based on the relative abundance of Q33 reads (and assuming sizes of all other phage genomes equate to the size of Q33 (31.1 kb)) indicate 9.92 ± 0.35 log_10_ viral particles g^− 1^ in sample 922, 10.14 ± 0.45 log_10_ viral particles g^− 1^ in sample 924 and 10.36 ± 0.40 log_10_ viral particles g^− 1^ in sample 925.

Where future samples are to be spiked with a single phage concentration, addition at a concentration of 10^6^ pfu g^− 1^ seems to be optimal in terms of percentage of reads aligning to the internal standard phage genome.

## Discussion

Since the first attempts of metagenomic analysis of viral communities in the gut by Breitbart and colleagues more than a decade ago [12], a considerable amount of sequence data has accumulated in public repositories. It has become clear that viral populations, although representing a smaller fraction of biomass and nucleic acid content in the faeces, actually match or even outnumber microbial cells in terms of copy numbers [19, 20]. Moreover, the viral component of the human gut microbiota appears to be dominated by unknown or uncharacterised viruses, which reach 60–95% of the total number of genotypes and are often being termed as ‘viral dark matter’ of the gut [19, 44]. However, even the annotated portion of gut viral genomic sequences published so far consists mainly of viruses that have never been cultured in the lab: prophages identified as parts of sequenced bacterial genomes, or extremely widespread and highly abundant viruses such as crAssphage, which had been described based solely on metagenomic sequence data [24, 51].

Presumably, most gut viruses are dsDNA bacteriophages belonging to the order *Caudovirales*, although phages and eukaryotic viruses with RNA, ssDNA and dsDNA genomes are also frequently detected [3, 19, 23, 26, 52]. It should be noted however that current knowledge of human gut phageomes is almost exclusively derived from studies conducted on faecal samples, and much less is known about the architecture of viral communities in the upper colon and small intestine [17, 18].

Despite a very limited knowledge existing about the physiological roles and ecological strategies of human gut phageomes, it has been suggested that the majority of gut viruses are temperate bacteriophages [21]. This is further supported by the observation of slow evolution and long-term temporal stability of the majority of viral genomes in longitudinal studies of human gut phageomes [23, 26]. In terms of phage-host interactions and populations dynamics, the human gut represents a very different type of environment when compared to the relatively better-understood phage marine ecosystems, where predator-prey interaction between bacteria and their viral parasites and ‘kill-the-winner’ effects play an important role in biomass control, regulation of microbial biodiversity and global biogeochemical cycles [21, 53].

At present, it is unclear how stable bacteriophage communities in the gut are assembled and what mechanisms underpin their temporal stability and inter-individual variability [22, 24]. It is unknown whether diverse phage populations of the gut play an active role in controlling the composition and density of bacterial populations or simply reflect the composition of the bacterial component of the microbiome. It was observed, however, that genetically dissimilar individuals living in the same household share certain portions of their gut phageomes [26]. Moreover, certain disease-specific phageome abnormalities often linked to the specific alterations of gut bacteriome have been reported in a number of human diseases [26–29].

In the present study, we have focused on some of the methodological aspects of metagenomic analysis of the viral ‘dark matter’ of the human gut. We describe a robust and relatively simple human faecal viral nucleic acid extraction protocol where the number of steps, overall complexity and hands-on time has been reduced to a reasonable minimum without significantly compromising the purity and yields of extracted viral nucleic acids.

Unlike many other previously published studies [21, 22, 24, 54, 55] (see Additional file 1 : Table S1 for details), we refrained from the use of CsCl gradients for purification of viral particles since this technique is labour intensive and poorly suited for high-throughput studies. Instead, we relied on a combination of microfiltration, PEG-precipitation of viral particles and removal of free nucleic acids by combined DNase/RNase treatment.

Total nucleic acid yields obtained using this protocol varied across a wide range, which can potentially reflect the wide variation of total viral loads in human samples (Additional file 2: Table S2). Levels of bacterial DNA contamination assessed roughly through percentage of reads aligned to bacterial rRNA databases also varied greatly, with a median value of 0.04%. Assuming a typical gut bacterial symbiont genome size of ~ 4 Mb and a ~ 5 kb rRNA operon present in 4 copies per genome, this level of rRNA gene contamination corresponds to only ~ 8% of DNA being of bacterial chromosomal origin. However, in some of the samples, levels of bacterial DNA contamination were, for unknown reasons, much higher with rRNA gene counts, reaching 0.47% of the reads in one sample (corresponding to close to 100% of bacterial DNA).

When levels of bacterial DNA contamination was assessed through alignment of reads to *cpn60* gene database, the median fraction of reads corresponding to the most conserved 549–567 bp segment within *cpn60* was 0.00077%. With assumptions similar to what we used in the above calculations for the rRNA gene, this corresponds to a median level of ~ 5.5% of bacterial genomic DNA in a sample. Interestingly, despite the overall moderate correlation (Pearson *r* = 0.469) between rRNA and *cpn60* alignment rates, the samples that demonstrated unusually high levels of rRNA reads (0.33 and 0.47%) had average *cpn60* levels (0.0036 and 0.00093%, respectively).

Additionally, relative abundances of rRNA and cpn60 reads from our faecal virome samples were compared to those from a selection (*n* = 18) of total faecal metagenomic samples sequenced as part of Human Microbiome Project. These are mostly composed of bacterial genomic DNA. As expected, median *cpn60* values from individual experiments within this study were significantly lower than median *cpn60* value for the HMP dataset (Additional file 3: Figure S1), with none of the virome samples surpassing the lowest *cpn60* value from the HMP samples. Surprisingly, with rRNA data, we observed that not only did a number of virome samples have alignment rates overlapping with those of the HMP dataset, but that some virome samples had *cpn60* levels even surpassing the highest values from the HMP dataset (Additional file 3: Figure S1). One possible explanation for this discrepancy and the striking over-representation of rRNA genes in some virome samples is that in those samples, large amounts of bacterial rRNA molecules could be introduced into nucleic acid samples by co-purification of ribosomes with viruses, with subsequent reverse transcription of rRNA into cDNA. Therefore, we believe that the conserved segment of *cpn60* gene can represent a viable alternative to rRNA as a marker of bacterial DNA contamination in faecal virome samples.

Meta-analysis conducted by Roux et al. [31] demonstrated presence of significant amounts of contaminating bacterial DNA in a quarter of viral metagenomes included in their study. It was recommended by the authors that levels of contamination corresponding to > 0.02% of rRNA reads should be considered as non-negligible and results from such samples should be treated with caution. At the same time, it seems that in practice, a certain level of bacterial DNA contamination in phageomes is inevitable even in studies where viral fractions are purified through CsCl density gradient centrifugation. Several studies have to date discussed that contamination with bacterial DNA can sometimes lead to considerable misinterpretations of sequencing data [56, 57]. Therefore, in the present study, we applied a number of in silico steps for removal of possible bacterial genomic sequences from our dataset at the expense of leaving behind ~ 50% of reads.

With a few notable exceptions [3, 58, 59], most of the studies of human gut phageomes published thus far have focused on DNA viruses and any RNA-containing viral species were neglected. In an early small-scale targeted metagenomic study of RNA viruses in the human gut, it was shown that the majority were plant viruses, with pepper mild mottle virus (PMMV, family *Virgaviridae*) being the most abundant [52]. Another study demonstrated acute presence of RNA bacteriophages in the non-human primate gut [60]. Interestingly, none out of 8920 contigs assembled in our study represented any known RNA viruses. Even so, a small proportion of individual reads in some samples could be aligned to plant RNA virus genomes, mostly to PMMV, suggesting that certain amounts of viral RNA was indeed co-purified with DNA and that the reverse transcription step was successful (data not shown). However, our attempt to artificially spike faecal samples with an RNA bacteriophage at three different concentrations (phage Qβ of the family *Leviviridae*) did not result in recovery of any reads aligning to its genome. This suggests that although some larger rod-shaped plant RNA viruses of family *Virgaviridae* were detected using our protocol, the protocol failed to quantitatively recover the smaller icosahedral, RNA-containing bacteriophage Qβ.

Similar to previous reports [24], the vast majority of contigs assembled from the data generated in this study (8759 out of 8920) could not be aligned to any known viral genomes in NCBI RefSeq database. Interestingly, a distinct separation of complete circular viral contigs into several size classes can be seen in Fig. 1b. A large group of contigs with sizes ranging from 4.7 to 6.8 kb carried gene complement characteristic of the *Microviridae* family (more specifically subfamilies *Gokushovirinae* and *Alpavirinae*) of ssDNA bacteriophages, the prevalence of which in the human gut has already been well documented [49, 61]. A group of circular contigs with intermediate sizes (38 to 46 kb) mostly consisted of temperate members of the phage families *Siphoviridae* and *Podoviridae* of the order *Caudovirales*, which together with the family *Microviridae* have been reported to be the most abundant groups of gut bacteriophages in three geographically distinct cohorts of IBD patients [26]. Another sizeable group of circular contigs, 94–101 kb long, shared a moderate degree of similarity in amino acid sequence of certain encoded proteins, as well as overall genome organisation and gene order, to the highly prevalent, but as yet uncultured, gut bacteriophage crAssphage [51]. Preliminary observations by Manrique et al. [24] as well as our own data suggests that crAssphage, rather than being an orphan evolutionary branch, might in fact be the first described representative of an unknown larger taxonomic group of gut bacteriophages. Lastly, representatives of a group of the shortest circular contigs (1–3 kb) had similarities (at nucleotide or amino acid sequence levels) to various short cryptic plasmids found in diverse taxonomic groups of bacterial gut symbionts. As previously discussed [35, 62], MDA amplification can introduce a significant bias into metagenomic analysis of complex phageome samples by favouring amplification of short single-stranded circular DNA molecules, such as single-stranded intermediate forms of rolling-circle replicating plasmids or small circular ssDNA viruses. The latter include members of *Microviridae*, *Anelloviridae*, *Circoviridae* families of bacteriophages and eukaryotic viruses commonly detected in metagenomic studies of human and animal gut phageomes, often after an MDA amplification step [3, 23, 26, 63]. Further improvements in sequencing library preparation techniques are essential to overcome this bias and to bring metagenomic research of human gut phageome to a fully quantitative level [35].

Of the contigs with sufficient level of sequence similarity to known viruses, only 4 were of non-bacteriophage (eukaryotic) origin. Two of them were HPV16 and HPV53 (family *Papillomaviridae*) present in samples from 6 individuals. Previously, presence of α– and β–genera of HPV was documented using PCR in 12.6% of samples from an Italian cohort of diarrhoea patients [64] and also was observed in sewage and sewage sludge [65]. The remaining two contigs included the plant virus TYLCV (family *Geminiviridae* of small circular ssDNA viruses) as well as an uncharacterised sewage-associated gemycircularvirus-4 (family *Genomoviridae* of small circular ssDNA viruses) believed to be specific towards an unknown fungal host [48], both with small circular ssDNA genomes.

It has been well established in microbiome research that variations in collection, storage and handling conditions for human faecal samples can significantly impact the outcome of microbiota profiling. With respect to sample collection protocols, rapid freezing of samples at − 80 °C is commonly considered as the best practice [37, 39]. However, for reasons of practicality, this can be replaced with storage at room temperature for up to 24 h [36], refrigeration or storage in a domestic freezer for up to 72 h [36–38]. Use of preservatives such as RNALater of Tris-EDTA was not recommended by the same studies. Different studies, however, often produce conflicting results regarding the effect of various storage conditions. In addition, it was reported that sample homogenisation by bead-beating, as well as repeated freeze-thaw cycles can lead to significant alterations in microbial profiles [40]. The latter observation is especially important with respect to some studies of the gut microbiome and phageome performed retrospectively, with archived faecal samples that had originally been collected for an alternative purpose. Also, strong between-lab alterations in 16S rRNA-based faecal microbiome profiles were reported in a recent study [66]. To the best of our knowledge, the effects of these and other potential confounding factors have never been investigated before with respect to human gut phageome profiling.

Previous literature indicates that room temperature storage for up to 24 h and even longer does not influence the number of observed bacterial taxa in faecal samples but can impact the composition and relative proportions within a sample [36]. Our results support this observation for both room temperature and + 4 °C and for both 16S rRNA bacteriome and phageome data. The amount of time the sample is stored for explains a similar amount of variation in both 16S and phageome profiles (14.2 and 10.9%, respectively). However, the magnitude of within-subject alterations due to storage procedures and hence the extent of instability was significantly lower in phageome profiles compared to bacteriome profiles. The latter observation can perhaps be explained by the nature of viral particles, their inherent stability in semi-liquid environments like faeces and relative insensitivity to biochemical stress factors (nutrient starvation, oxidative stress, action of organic and bile acids).

Consistent with previous literature data [39, 40], a single or repeated (up to 5) freeze-thaw cycles did not cause any significant alterations of faecal bacterial community structure as assessed by 16S rRNA profiling. A very similar result was observed for the phageome data. Interestingly, analysis of α-diversity showed a certain trend for increase in richness (Shannon entropy index) of the bacteriome with increasing number of freeze-thaw cycles (as demonstrated in Additional file 4: Figure S2a). Such an effect could possibly be explained by an assistive action of repeated freeze-thaw cycles on efficiency of DNA extraction from difficult-to-lyse bacteria (*Actinobacteria*, *Firmicutes*) which leads to an increase in visible richness and evenness of bacteriome composition. No such trend could be seen in the corresponding faecal phageome dataset. This may result from the fact that many viruses survive freeze-thaw cycling [67], and efficiency of nucleic acid extraction from different types of viruses does not differ to the extent it does among certain Gram-positive and Gram-negative bacteria.

Another significant effect was in operator-to-operator differences in VLP DNA extraction from faecal samples. Unlike 16S rRNA gene-based profiling of bacteriomes, where operator effect seemed to be negligible, variation in phageome profiling was significant (2.8% of total variation in the data sets) and can explained by the operator variable. This difference in operator impact on bacteriome versus phageome data is most probably a consequence of the more elaborate and complicated procedure required for VLP DNA extraction. Such processing requires in-house preparation of reagents and includes multiple manual steps that are vulnerable to potential systematic or random operator mistakes. In comparison, total DNA extraction from faeces was performed entirely using a commercially available kit-based protocol with little space for operator error. We believe that although the magnitude of the operator effect was not considerable, it still highlights the necessity of strict standardisation in faecal phageome profiling, especially in large scale comparative and longitudinal studies.

As previously mentioned, published data on the absolute concentrations of VLPs in human faecal and mucosal samples are often conflicting and highly dependent on the extraction procedure and method of quantification [17, 18, 20]. To the best of our knowledge, none of the published metagenomic studies have attempted to assess viral loads quantitatively or semi-quantitatively by spiking faecal samples with an exogenous virus as an internal standard. In our hands, introduction of an exogenous internal phage standard provided a promising tool for estimating total viral loads in human faecal samples. In the three faecal samples tested in this study, the estimated total viral concentrations ranged from 9.92 ± 0.35 to 10.36 ± 0.40 log_10_ viral particles g^− 1^.

## Conclusions

The overall effect observed in the present study was a greater relative individual specificity of phageome profiles obtained using shotgun metagenomic sequencing of faecal VLP fractions compared to bacteriome profiles obtained using amplicon sequencing of 16S rRNA gene fragments. This was not completely surprising since shotgun sequencing of total microbial DNA is known to produce a snapshot of microbiota at a much higher taxonomic resolution compared to amplicon sequencing of highly conserved fragments of 16S rRNA genes [68, 69]. In our hands, faecal phageomes and bacteriomes seemed to react similarly to being stored at + 4 °C or room temperature. However, phageome profiles were less affected by multiple freeze-thaw cycles than 16S rRNA bacteriome profiles. Importantly, there was a greater chance for operator effect in the processing of phageomes compared to bacteriomes. Taken together, the above results suggest that the biological signal of faecal virome composition generated using our optimised protocol from each of the subjects was consistently and significantly stronger than the signal from any technical factor we tested. The spiking of faecal samples with exogenous DNA bacteriophage was successful, and as a result, we were able to see a large variation in the total viral load, even though only a small number of individuals were tested. In addition to that, numerous novel phage sequences were observed in this study. Of special interest was identification of several ~ 100 kb bacteriophage genomes containing genomic features homologous to highly predominant but poorly characterised crAssphage.

## Methods

### Sample collection and storage

Faecal samples were collected from consenting volunteers according to study protocol APC055, approved by the Cork Research Ethics Committee (CREC). The samples were collected (without fixative or preservative) in the volunteer’s home and transported to the research facility at ambient temperature, avoiding exposure to heat. They were generally stored at − 80 °C until processed, unless indicated otherwise.

### Storage experiment

Fresh faecal samples were collected from four healthy adults. Immediately after delivery, these were divided into 0.5 g aliquots. One tube from each sample was frozen immediately at − 80 °C. One tube of each sample was stored at + 4 °C for 6 h and the other for 24 h. Two additional tubes were incubated at room temperature and collected at 6 and 24 h. Once the desired end point was reached, the samples were rapidly frozen at − 80 °C for subsequent VLP and total faecal DNA extraction as described below.

### Freeze-thaw experiment

Fresh faecal samples were collected from three healthy adults, aliquoted immediately upon receipt and frozen at − 80 °C. One aliquot of each sample was kept frozen for 17 days. On five consecutive days, a daily freeze-thaw cycle was carried out on each of the samples by leaving the sample for 45 min at room temperature with subsequent freezing at − 80 °C. Aliquots removed from thawed samples on day 3 and day 5 were subjected to VLP and total faecal DNA extraction, as were the aliquots stored for their entirety (17 days) at − 80 °C.

### Operator reproducibility experiment

Frozen faecal samples collected from 3 healthy adults and 3 IBD patients were processed twice on two separate days by each of the three operators. VLP and total DNA extractions were carried out following the protocols below.

### Spiking of faecal samples with lactococcal phage Q33 and coliphage Qβ

Lactococcal phage Q33 [50] was propagated using strain *L. lactis* SMQ-86 as a host at 30 °C in M17 broth (Oxoid) supplemented with 0.5% glucose without agitation. Qβ was propagated using *E. coli* MG1655 as a host strain at 37 °C in LB broth with agitation. Phage lysates were filtered by passing through 0.45-μm pore membrane filters and stored at + 4 °C. Faecal samples collected from three healthy adult donors were split into 0.5 g aliquots, homogenised and then spiked with filtered Q33 and Qβ phage lysates to final concentrations of 10^5^, 10^6^ or 10^7^ pfu g^− 1^ of faecal sample. Faecal VLP nucleic acids were isolated as outlined below. Unspiked aliquots of the same samples were included as negative controls.

### Faecal VLP nucleic acid extraction

Aliquots of 0.5 g of faeces were resuspended in 10 mL of SM buffer and homogenised by vigorous vortexing for 5 min. Tubes were then chilled on ice for 5 min prior to centrifugation at 5000 rpm in a swing bucket rotor for 10 min at + 4 °C. Supernatants were transferred to new tubes, and centrifugation was repeated once again. Supernatant were subsequently filtered twice through a 0.45-μm pore PES syringe-mounted membrane filters. NaCl and PEG-8000 powders were then added to filtrates to give a final concentration of 0.5 M and 10% *w*/*v*, respectively, after complete dissolving samples were incubated overnight (16 h) at + 4 °C.

On the following day, the samples were centrifuged at 5000 rpm for 20 min at + 4 °C to collect precipitate. Supernatant was removed, and tubes were left in inverted position on paper towels for 5 min to remove last traces of supernatant. Pellets were then resuspended in 400 μl of SM buffer and extracted by gentle shaking with equal volume of chloroform. Emulsions were then centrifuged at 2500 g for 5 min using a desktop centrifuge. The aqueous phase (~ 360 μl) was aspirated into clean Eppendorf tubes and mixed with 40 μl of a solution of 10 mM CaCl_2_ and 50 mM MgCl_2_. After addition of 8 U of TURBO DNase (Ambion/ThermoFisher Scientific) and 20 U of RNase I (ThermoFisher Scientific) free DNA/RNA digestion was carried out at 37 °C for 1 h before inactivating enzymes at 70 °C for 10 min. Proteinase K (40 μg) and 20 μl of 10% SDS were then added and to the tubes, and incubation was continued for 20 min at 56 °C. Finally, viral particles were lysed by addition of 100 μl of Phage Lysis Buffer (4.5 M guanidinium isothiocyanate, 44 mM sodium citrate pH 7.0, 0.88% sarkosyl, 0.72% 2-mercaptoethanol) and incubation at 65 °C for 10 min. Lysates were then extracted twice by gentle vortexing with equal volume of Phenol/Chloroform/Isoamyl Alcohol 25:24:1 (Fisher Scientific) followed by centrifugation at 8000 g for 5 min at room temperature. The resulting aqueous phase was subjected to final round of purification using DNeasy Blood & Tissue Kit (Qiagen) according to manufacturer’s instruction with a final elution volume of 50 μl.

### Shotgun sequencing of faecal VLP nucleic acids

Twelve microliters of faecal VLP nucleic acid sample regardless of concentration was taken into reverse transcription reaction using SuperScript IV Reverse Transcriptase (RT) kit (Invitrogen/ThermoFisher Scientific) according to the manufacturer’s random hexamer primer protocol. One microliter of reverse-transcribed nucleic acids was then amplified using MDA technology with Illustra GenomiPhi V2 kit (GE Healthcare). The latter step was done in triplicate for each sample. Products from all three MDA reactions, together with the remainder of RT products (17 μl), were pooled together and subjected to additional round of purification using DNeasy Blood & Tissue Kit.

Amplified DNA was quantified using Qubit dsDNA HS Assay Kit (Invitrogen/ThermoFisher Scientific) and subjected to random shotgun library preparation using Nextera XT DNA Library Preparation Kit (Illumina) and bead-based normalisation following the standard manufacturer’s protocol. Ready-to-load libraries were sequenced using a proprietary modified protocol using 2 × 300 bp paired-end chemistry on an Illumina MiSeq platform (Illumina, San Diego, California) at GATC Biotech AG, Germany.

### Total faecal DNA extractions and library preparation for microbiota profiling using 16S rRNA amplicon sequencing

The QIAamp Fast DNA Stool Mini Kit (Qiagen, Hilden, Germany) was used according to manufacturer’s guidelines to extract total faecal DNA from ~ 200 mg aliquots of faeces, but was modified to include a bead-beating step. The samples were placed in 2 mL screw-cap tubes containing 1 mL of InhibitEX Buffer and a mixture of inert beads (ThistleScientific) of various diameters (one 3.5 mm glass bead, ~ 200 μl of 1 mm zirconium beads, ~ 200 μl of 0.1 mm zirconium beads). Following 2× 30s beating in FastPrep-24 instrument (MP Biomedicals) with an intermittent step of cooling on ice for 30 s, the samples were lysed by heating for 5 min at 95 °C. Subsequently, the samples were processed according to the standard Qiagen protocol.

Hypervariable regions V3 and V4 of bacterial 16S ribosomal RNA genes were amplified from 15 ng of total DNA template via PCR using Phusion High-Fidelity PCR Master Mix (ThermoFisher Scientific) and 0.2 μM of each of the primers 16S-FP: 5′-TCGTCGGCAGCGTCAGATGTGTATAAGAGACAGCCTACGGGNGGCWGCAG-3′ and 16S-RP: 5′-GTCTCGTGGGCTCGGAGATGTGTATAAGAGACAGGACTACHVGGGTATCTAATCC-3′ containing the appropriate Illumina Nextera XT overhang adapter sequences (sequence portions complementary to bacterial 16S rRNA genes are underlined). The following PCR program was used: 98 °C 30s, 25 cycles of 98 °C 10s, 55 °C 15 s, 72 °C 20s, final extension 72 °C 5 min. Following purification using Agencourt AMPure XP magnetic beads (Beckman-Coulter), the amplicon libraries underwent a second PCR reaction to attach dual Illumina Nextera indices using the Nextera XT index kit v2 (Illumina). Following purification (as described above), the dsDNA libraries were quantified using a Qubit dsDNA HS Assay Kit and pooled in equimolar concentrations. Ready-to-load libraries were sequenced using a proprietary modified protocol using 2 × 300 bp paired-end chemistry on an Illumina MiSeq platform (Illumina, San Diego, California) at GATC Biotech AG, Germany.

### Analysis of 16S rRNA amplicon sequencing data

The quality of the raw reads was visualised with FastQC v0.11.3. The reads were then imported into R v3.3.0 for analysis with the DADA2 package (v1.03) [70]. Errors introduced during the sequencing process were corrected to generate ribosomal sequence variants (RSVs). These were exported and further chimera filtered using both the de novo and reference-based chimera filtering implemented in USEARCH v8.1.1861 with the ChimeraSlayer gold database v20110519 [71]. The remaining RSVs were classified with mothur v1.38 [72] against the RDP database version 11.4, as well as classified with SPINGO to species level [73]. Only RSVs with a domain classification of bacteria or archaea were kept for further analysis. A phylogenetic tree of the RSV sequences rooted on the midpoint was generated with FastTree [74].

### Analysis of phageome shotgun sequencing data

The quality of the raw reads was visualised with FastQC v0.11.3. Nextera adapters were removed with cutadapt v1.9.1 [75] followed by read trimming and filtering with Trimmomatic v0.36 [76] to ensure a minimum length of 60, maximum length of 250 and a sliding window that cuts a read once the average quality in a window size of 4 follows below a Phred score of 30. Reads were then assembled on a per sample basis with the metaSPAdes assembler [77]. Redundancy between samples was removed by aligning all contigs against each other with BLAST+ v2.2.28. Only the longer contig was kept in the reference set when contigs aligned to each other with at least 90% identity over 90% of their length. In order for a contig to be included in the final analysis, it must have been detected as viral by Virsorter [44] in the phageome decontamation mode, or had a significant BLAST hit (50% identity over 90% of the length) to a genome in RefSeq Virus [45, 46], or had no significant BLAST hits (alignments longer than 100 nucleotides with an *e* value greater than 1e−5) against the ‘nt’ database. This allowed us to include known viruses, putative viruses predicted by Virsorter and completely novel viral sequences not yet included in any database. The quality-filtered reads were then aligned to this contig set using bowtie2 v2.1.0 [78] using the end-to-end alignment mode. A count table was generated with samtools v0.1.19 which was then imported into R v3.3.1 for statistical analysis. Levels of bacterial DNA contamination were estimated by classifying reads with SortMeRNA v2.0 [79] against the SILVA database [80] to assess 16S rRNA abundance and by aligning reads using bowtie2 (*--very-sensitive* preset mode) against the cpnDB [43] to assess abundance of cpn60 gene. A selection of total faecal DNA metagenomic samples (sample IDs SRS012902, SRS013158, SRS013215, SRS014613, SRS014923, SRS015854, SRS016095, SRS016753, SRS017521, SRS018575, SRS018656, SRS019601, SRS020328, SRS022137, SRS024549, SRS045645, SRS053398, SRS054956) deriving from Human Microbiome Project was used for comparisons.

### Statistical analysis

Alpha diversity and beta diversity were generated using PhyloSeq v1.16.2, which also was used for a principle coordinate analysis as implemented in Ape v3.5. As gut microbiome datasets typically do not follow a normal distribution, the non-parametric Mann-Whitney *U* test was implemented for assessing differences between two groups, and the non-parametric Kruskal-Wallis test where there are more than two groups. Differential abundance analysis was carried out with DESeq2 v1.12.4 [81], an algorithm originally designed for RNA-Seq analyses but has found to be efficient in microbiome datasets [82]. All visualisation in *R* was performed with ggplot2 v2.2.1. The Adonis function in the Vegan library v2.4.3 was used to look for associations with metadata factors. This implements a permutational multivariate analysis of variance (PERMANOVA), which allows for identifying associations between distance matrices and meta data variables assigning significance through a permutation-based test [83].

## Additional files

Additional file 1: **Table S1.** Comparison of methods used in published metagenomic studies of viral communities associated with various sites in the human body. (XLSX 14 kb)

Additional file 2: **Table S2.** Description of faecal samples used in the study and summary of nucleic acid extraction and sequencing results. (XLSX 161 kb)

Additional file 3: **Figure S1.** Percentage of reads aligned to conserved segment of bacterial *cpn60* gene and rRNA genes operon in virome samples from four separate experiments within this study compared to a selection of 18 total metagenomic samples sequenced as part of the Human Microbiome Project. (PDF 29 kb)

Additional file 4: **Figure S2.** Effect of storage on the composition of faecal phageomes and bacteriomes (continued from Fig. 2 in the main text). A and B, α-diversity of bacterial (16S) and viral populations in faecal samples stored at two different temperatures for up to 24 h. C and D, Spearman rank ecological distances (β-diversity) between stored aliquotes of the 4 faecal samples used in the experiment, calculated for both bacterial 16S and viral datasets and visualised via non-metric multidimensional scaling (NMDS); NMDS stress values were 0.1332468 (C) and 0.09051503 (D). E and F, Barplots of relative abundance of bacterial taxonomic groups at the genus level and viruses at the individual contig level. (PDF 393 kb)

Additional file 5: **Table S3.** DESeq2 test results for differential abundance of putative phage contigs in six faecal samples processed three different operators (O1, O2 and O3). (XLSX 730 kb)

Additional file 6: **Figure S3.** Effect of repeated freeze-thaw cycles on the composition of faecal phageomes and bacteriomes (continued from Fig. 3 in the main text). A and B, α-diversity of bacterial (16S) and viral populations in faecal samples subjected to up to 5 successive freeze-thaw cycles or a prolonged storage at − 80 °C. C and D, Spearman rank ecological distances (β-diversity) between aliquotes of the three faecal samples used in the freeze-thaw experiment, calculated for both bacterial 16S and viral datasets and visualised via NMDS; NMDS stress values were 9.274361e-05 (C) and 9.926038e-05 (D). E, Barplot of relative abundance of bacterial taxonomic groups at the genus level. (PDF 376 kb)

Additional file 7: **Figure S4.** Operator-dependent variations in faecal phageome and bacteriome profiling results (continued from Fig. 4 in the main text). A and B, α- diversity of bacterial (16S) and viral populations in six faecal samples processed for nucleic acids extraction twice by each of the three operators. C and D, Spearman rank ecological distances (β-diversity) between aliquotes of the six faecal samples used in the experiment, calculated for both bacterial 16S and viral datasets and visualised via NMDS. NMDS stress values were 0.08977516 (C) and 0.2412623 (D). E and F, Barplots of relative abundance of bacterial taxonomic groups at the genus level and viruses at the individual contigs level. (PDF 426 kb)

Additional file 8: **Table S4.** Details on Adonis (PERMANOVA) test output. (XLSX 11 kb)

## Abbreviations

GIT: Gastro-intestinal tract, gut microbiome
HPV: Human papillomavirus
HTS: High-throughput sequencing
IBD: Inflammatory bowel disease
ICTV: International committee on taxonomy of viruses
MDA: Multiple displacement amplification
PEG: Polyethylene glycol
PES: Polyethersulfone
PMMV: Pepper mild mottle virus
RSV: Ribosomal sequence variant
TFF: Tangential flow filtration
TYLCV: Tomato yellow leaf curl virus
VLP: Virus-like particle
WGA: Whole-genome amplification
