# Supplementary figures and images for: Reproducible protocols for metagenomic analysis of human faecal phageomes
Source: Microbiome. 2018 Apr 10;6:68. doi: 10.1186/s40168-018-0446-z (PMC5892011; doi:10.1186/s40168-018-0446-z)

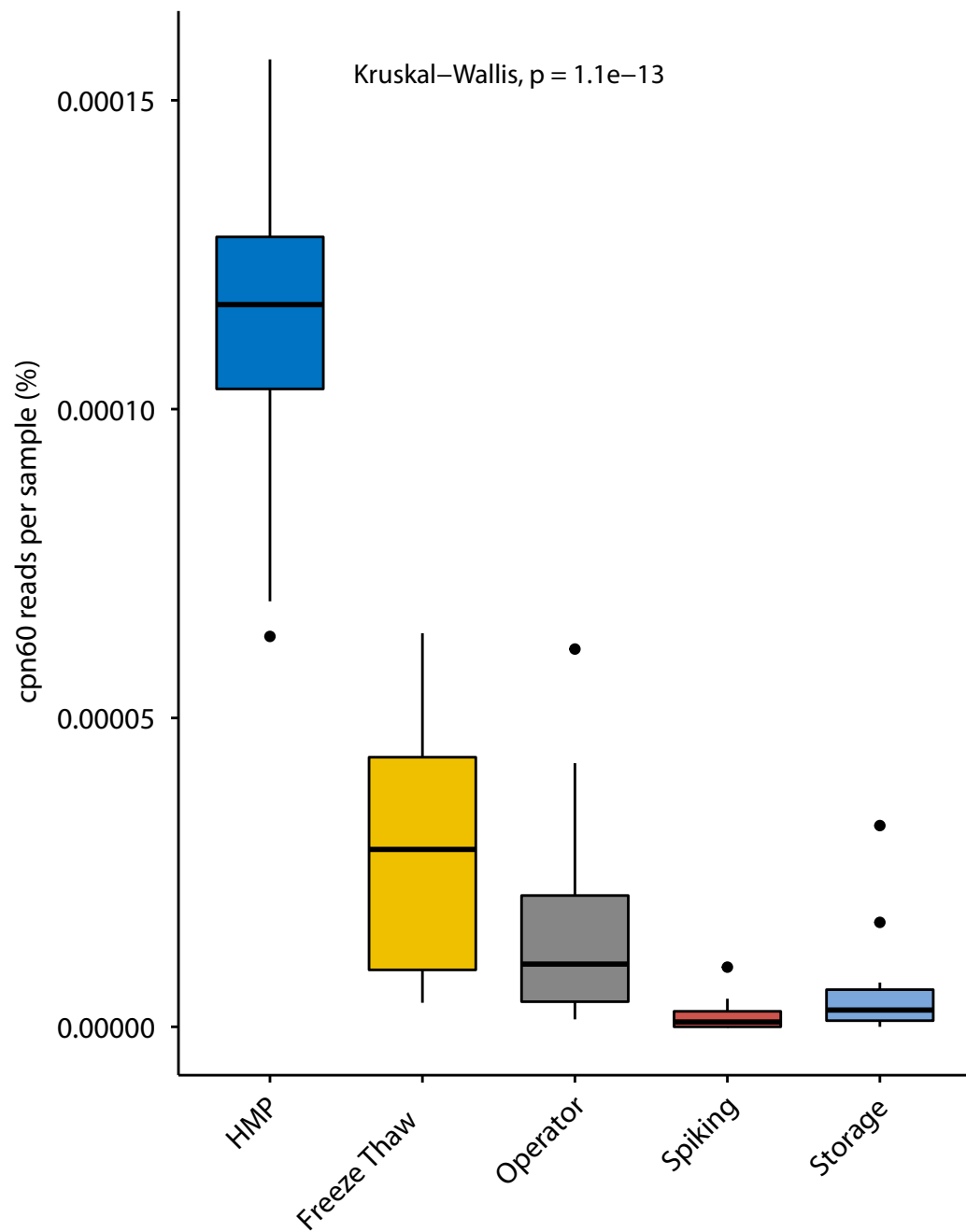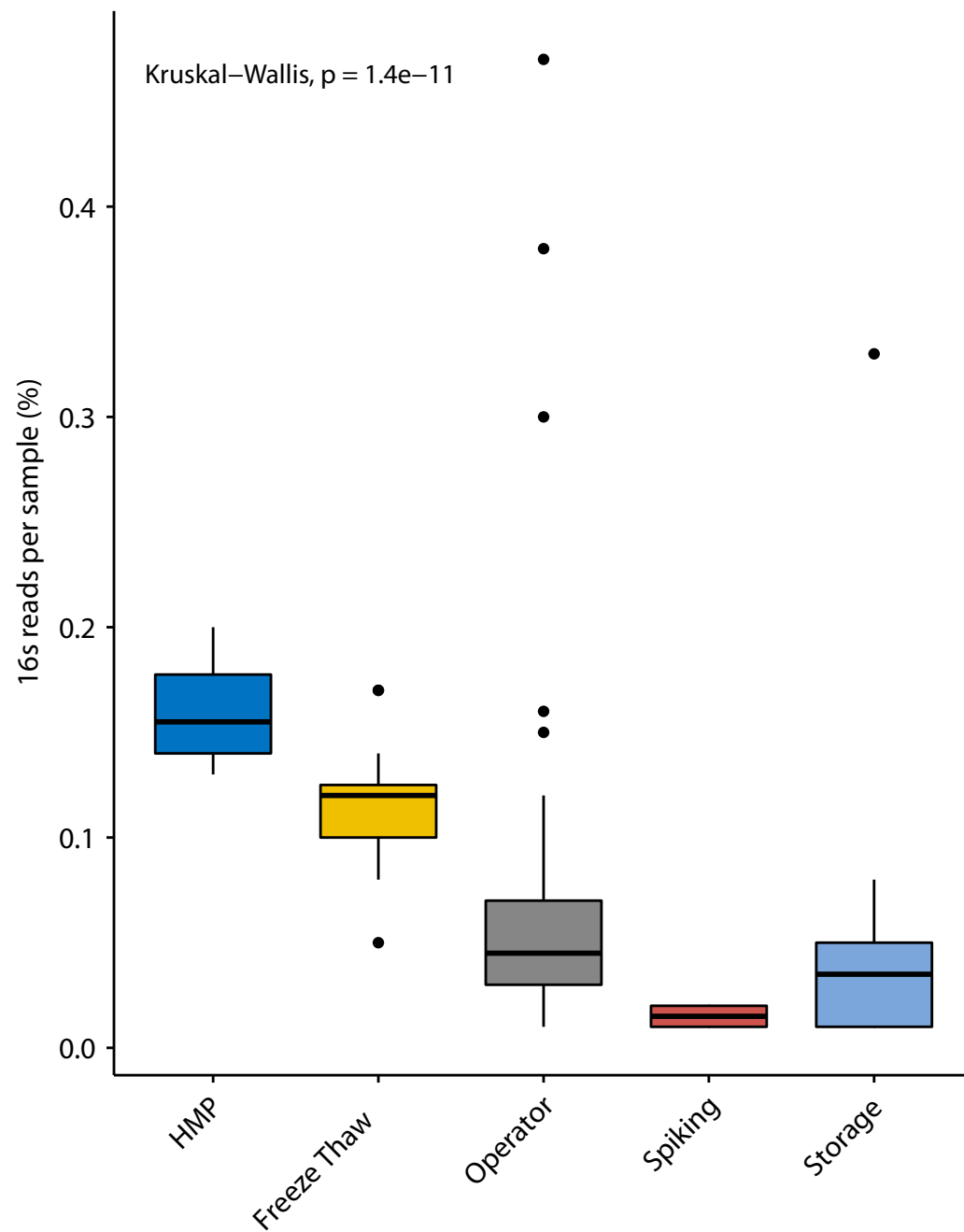

Supplement: Supplementary file 3 — Figure S1. Percentage of reads aligned to conserved segment of bacterial cpn60 gene and rRNA genes operon in virome samples from four separate experiments within this study compared to a selection of 18 total metagenomic samples sequenced as part of the Human Microbiome Project. (PDF 29 kb) [file 40168_2018_446_MOESM3_ESM.pdf]

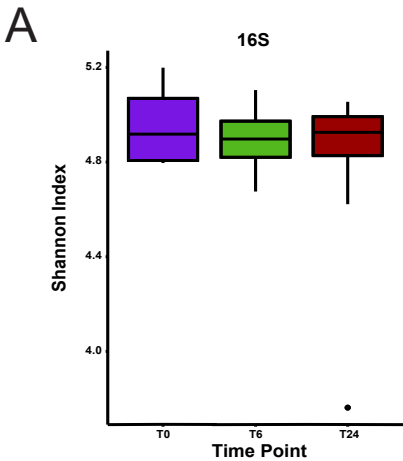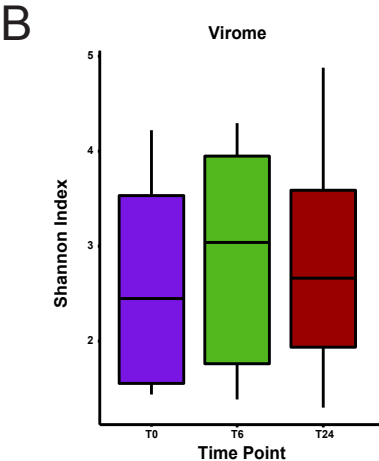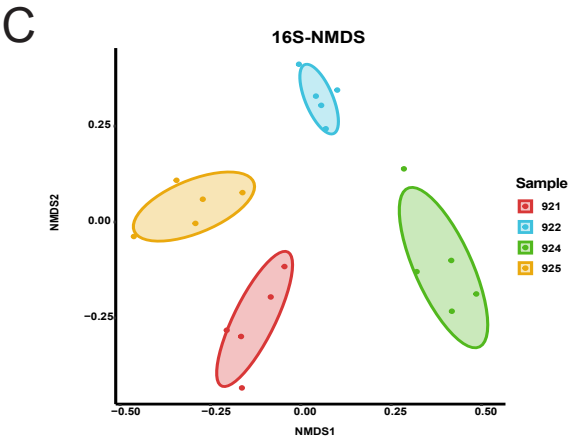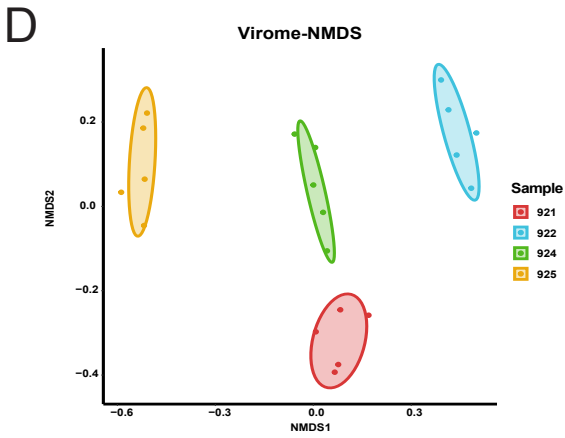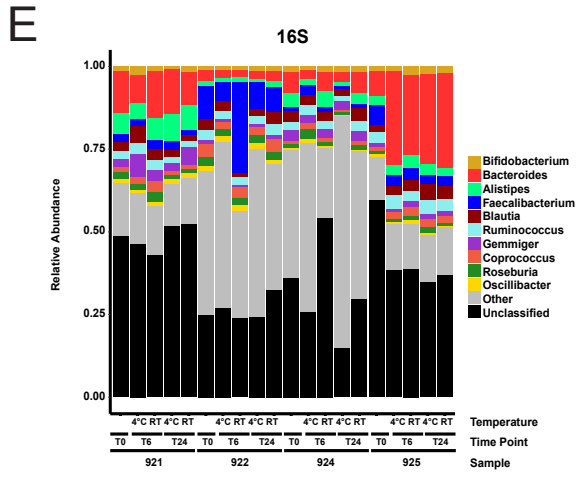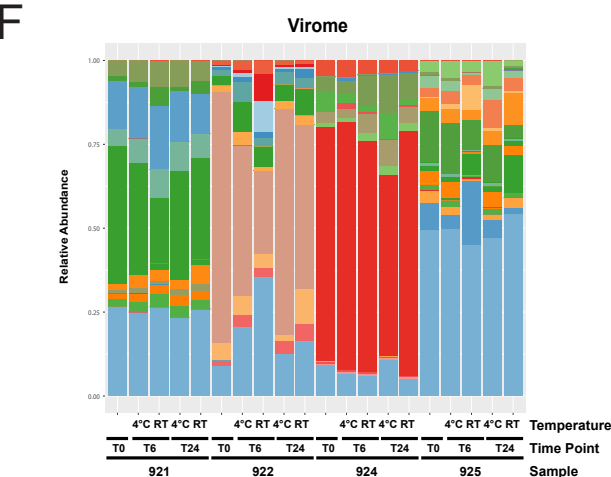

Supplement: Supplementary file 4 — Figure S2. Effect of storage on the composition of faecal phageomes and bacteriomes (continued from Fig. 2 in the main text). A and B, α-diversity of bacterial (16S) and viral populations in faecal samples stored at two different temperatures for up to 24 h. C and D, Spearman rank ecological distances (β-diversity) between stored aliquotes of the 4 faecal samples used in the experiment, calculated for both bacterial 16S and viral datasets and visualised via non-metric multidimensional scaling (NMDS); NMDS stress values were 0.1332468 (C) and 0.09051503 (D). E and F, Barplots of relative abundance of bacterial taxonomic groups at the genus level and viruses at the individual contig level. (PDF 393 kb) [file 40168_2018_446_MOESM4_ESM.pdf]

A

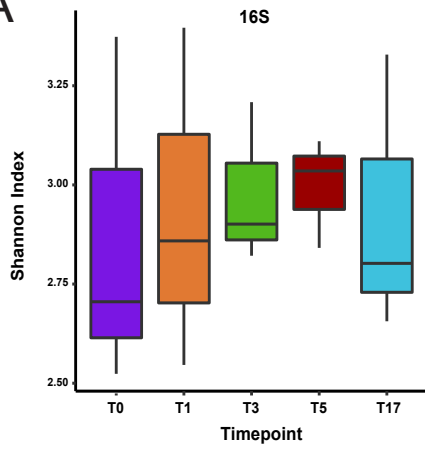

B

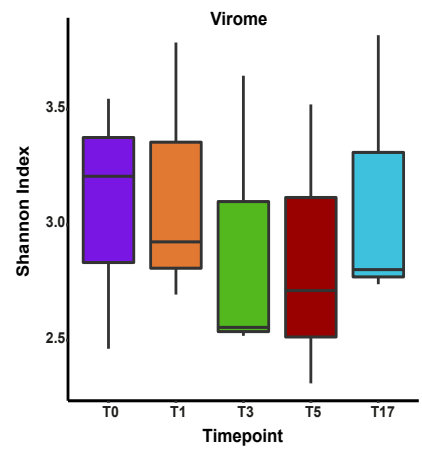

C

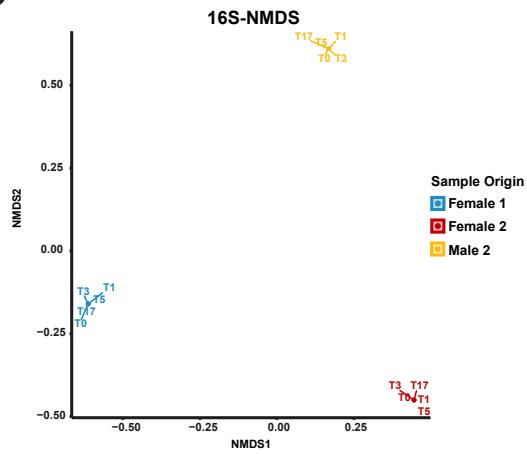

D

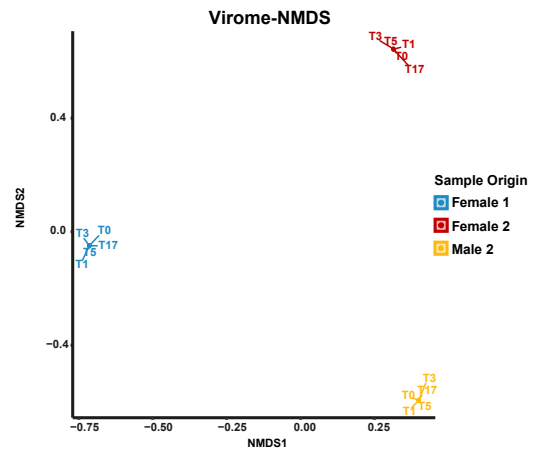

E

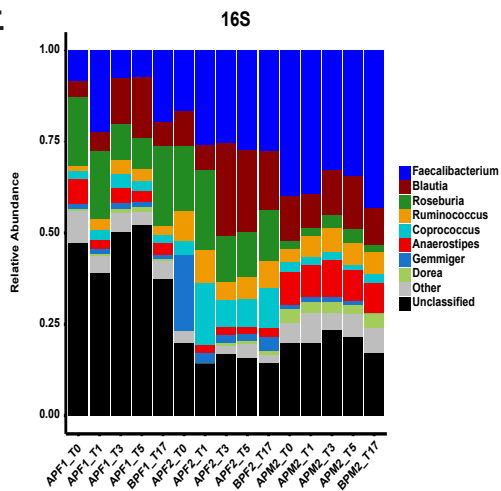

Supplement: Supplementary file 6 — Figure S3. Effect of repeated freeze-thaw cycles on the composition of faecal phageomes and bacteriomes (continued from Fig. 3 in the main text). A and B, α-diversity of bacterial (16S) and viral populations in faecal samples subjected to up to 5 successive freeze-thaw cycles or a prolonged storage at − 80 °C. C and D, Spearman rank ecological distances (β-diversity) between aliquotes of the three faecal samples used in the freeze-thaw experiment, calculated for both bacterial 16S and viral datasets and visualised via NMDS; NMDS stress values were 9.274361e-05 (C) and 9.926038e-05 (D). E, Barplot of relative abundance of bacterial taxonomic groups at the genus level. (PDF 376 kb) [file 40168_2018_446_MOESM6_ESM.pdf]

A

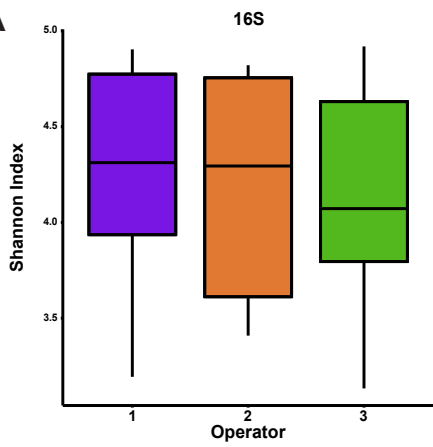

B

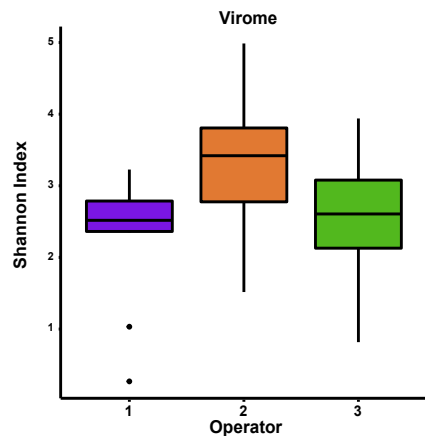

C

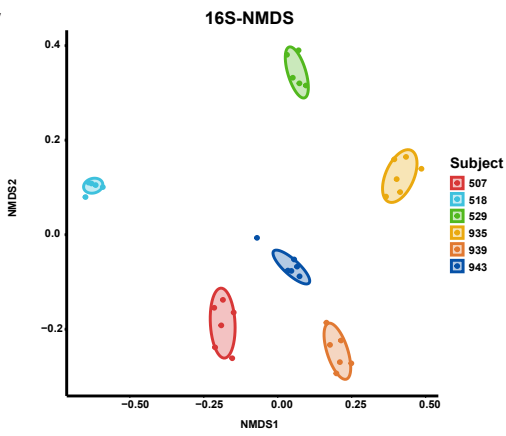

D

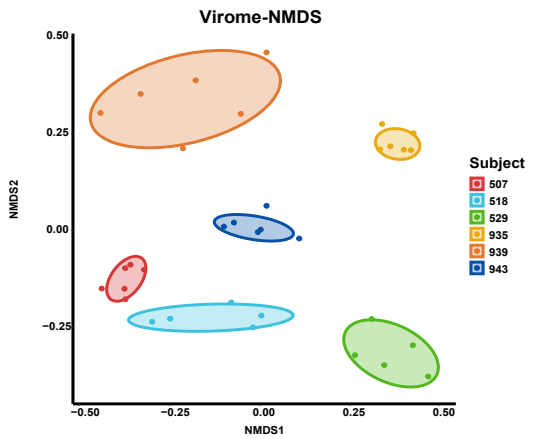

E

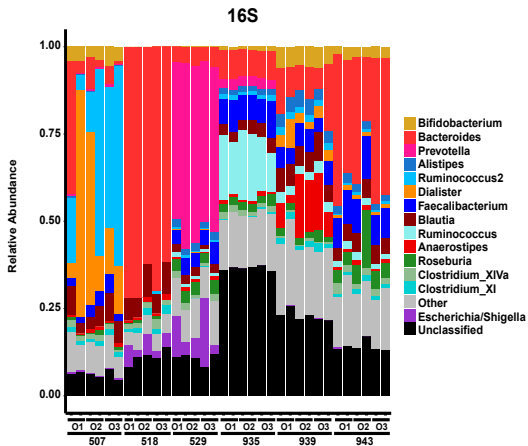

F

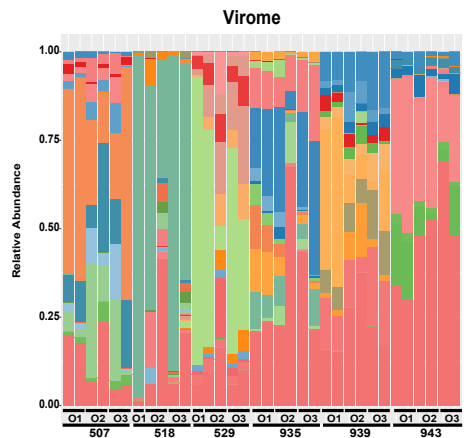

Supplement: Supplementary file 7 — Figure S4. Operator-dependent variations in faecal phageome and bacteriome profiling results (continued from Fig. 4 in the main text). A and B, α- diversity of bacterial (16S) and viral populations in six faecal samples processed for nucleic acids extraction twice by each of the three operators. C and D, Spearman rank ecological distances (β-diversity) between aliquotes of the six faecal samples used in the experiment, calculated for both bacterial 16S and viral datasets and visualised via NMDS. NMDS stress values were 0.08977516 (C) and 0.2412623 (D). E and F, Barplots of relative abundance of bacterial taxonomic groups at the genus level and viruses at the individual contigs level. (PDF 426 kb) [file 40168_2018_446_MOESM7_ESM.pdf]
